# The Inhibition of Caspase-1- Does Not Revert Particulate Matter (PM)-Induced Lung Immunesuppression in Mice

**DOI:** 10.3389/fimmu.2019.01329

**Published:** 2019-06-21

**Authors:** Chiara Colarusso, Gianluigi De Falco, Michela Terlizzi, Fiorentina Roviezzo, Ida Cerqua, Mariano Sirignano, Giuseppe Cirino, Rita P. Aquino, Aldo Pinto, Andrea D'Anna, Rosalinda Sorrentino

**Affiliations:** ^1^Department of Pharmacy (DIFARMA), University of Salerno, Fisciano, Italy; ^2^PhD Program in Drug Discovery and Development, Department of Pharmacy, University of Salerno, Fisciano, Italy; ^3^Department of Chemical, Material and Industrial Engineering, University of Naples “Federico II”, Naples, Italy; ^4^Department of Pharmacy, University of Naples “Federico II”, Naples, Italy

**Keywords:** combustion-generated ultrafine particles (UFPs), soot, lung inflammation, airway responsiveness, immunesuppression

## Abstract

**Background:** Air pollution is becoming a threatening issue for human health. Many epidemiological studies relate air pollution index to adverse effects in terms of disease incidence and/or disease exacerbation. In our previous studies, we found air pollutants can induce the release of pro-inflammatory cytokines from human peripheral blood cells. To better understand, the effects of air pollution in the lung, we took advantage of an animal model.

**Experimental Approach:** Mice were intratracheally and daily exposed to urban collected particulate matter (PM, PM10, and PM1) and to the sub-micrometric carbonaceous component, Soot.

**Results:** We found that PM10, PM1, and Soot promoted lung inflammation associated to higher bronchial responsiveness and lower dilation together with an immunosuppressive lung environment, characterized by tolerogenic dendritic cells (DCs), macrophages and myeloid -derived suppressor cells (MDSCs), the latter two Arginase I positive. In support, higher recruitment of Treg associated to higher levels of IL-10 were detected in the lung of PM10, PM1, and Soot treated mice. This effect was not abolished by the administration of a caspase-1 inhibitor, Ac-Y-VAD, implying that the canonical inflammasome complex was not associated to PMx-induced lung immunosuppression in mice.

**Conclusion:** Our study proves that PM exposure leads to an immunosuppressive lung environment in a caspase-1-independent manner, paving the way to understand the molecular and cellular mechanism/s underlying the establishment of some respiratory disorders according to the exposure to air pollution.

## Introduction

In the recent decades, air pollution is becoming a growing cause for concern of human health in that a strong correlation has been demonstrated between air pollution and a wide range of adverse effects/disease incidence/disease exacerbation (WHO 2019, https://www.who.int/airpollution/ambient/health-impacts/en).

Air pollutants are composed of particulate matter (PM), ozone (O_3_), nitrogen dioxide (NO_2_), nitrogen oxides (NOx), sulfur dioxide (SO_2_), and Volatile and Semivolatile Organic Carbon (VOC and SOC, respectively) (https://www.eea.europa.eu/publications/air-quality-in-europe-2018). PM directly emitted from anthropogenic activities (e.g., soot from combustion sources) or by natural factors (e.g., sea salt and soil from wind-driven suspension) is defined primary PM. Secondary PM is instead formed in the atmosphere through oxidation processes involving precursor pollutants (NOx, VOS and SOC, SOx) activated by solar radiation. PM can be characterized in terms of mass concentration of particles with smaller size than 2.5 μm (PM2.5) or 10 μm (PM10), in terms of number concentration of particles (essentially the ultrafine fraction, i.e., particles with smaller size than 100 nm), or in terms of the chemical composition (e.g., black carbon, organic compounds and heavy metals) (1). PM10 may be an appropriate indicator when considering the impact of resuspension of road dust, while black carbon is a more sensitive indicator of exhaust emissions from road traffic (2, 3). Epidemiological and toxicological evidence show that PM2.5 and PM10 comprise fractions with varying types and degrees of health effects (http://www.euro.who.int/__data/assets/pdf_file/0020/182432/e96762-final.pdf). In particular, in Europe premature deaths are 500.000, among which 428.000 are related to PM2.5 exposure (https://www.eea.europa.eu/publications/air-quality-in-europe-2017). Herein, these concepts are related to the entire world.

The direct link between air pollution and its impact on human health is represented by the respiratory tract, that allows ultrafine particles (UFPs, sub-100 nm particles) to settle down in the lower tract of the respiratory tree (4). Instead, larger PMs (>2.5 μm) tend to be trapped by the nose and upper airways, easily exhaled and thus at a lower concentration than the PM with smaller size or UFPs (5). Hence, it is nowadays well established that UFPs represent the most threatening particles in that they can localize into the low tract of the respiratory tree leading to pulmonary diseases (4). In this regard, we proved that UFPs, also defined as Soot, representative of those encountered in practical combustion conditions, including gasoline and diesel engines, induce pro-inflammatory pathways in smokers (6) and chornic pulmonary disease (COPD) patients (7).

In these latter studies, we found that Soot particles activate human peripheral blood cells, which then release pro-inflammatory cytokines (i.e., IL-1-like cytokines) after the activation of the multiprotein complex inflammasome. In the present study, instead, we took advantage of an animal model of PM exposure in order to understand the immune microenvironment in the lung. In particular, we found that urban collected PM (i.e., PM10 and PM1) and the sub-micrometric component, Soot, lead toward lung inflammation characterized by the recruitment of immunosuppressive cells, such as myeloid-derived suppressor cells (MDSCs) and M2 macrophages, which were Arginase I positive, and that were correlated to the presence of both anti-inflammatory cytokines (i.e., IL-10) and suppressive Treg, paving the way for some respiratory disorders (i.e., cancer).

## Materials and Methods

### Mice

Female specific pathogen-free C57BL/6 mice (6–8 weeks of age) (Charles River Laboratories, Lecco, Italy) were fed with a standard chow diet and housed under specific pathogen-free conditions at the University of Salerno, Department of Pharmacy. Female mice were chosen because less aggressive than males, avoiding any interference with the interpretation of the data. All animal experiments were performed under protocols that followed the Italian and European Community Council for Animal Care (2010/63/EU). This study was carried out in strict accordance with the recommendations in the Guide for the Care and Use of Laboratory Animals of the National Institutes of Health. The protocol was approved by the Committee on the Ethics of Animal Experiments of the University of Salerno and Health Ministry with the approval number 985/2017.

### Experimental Protocol

Mice were intratracheally (i.t.) instilled with Soot (90 ng/mouse), PM1 (25 ng/10 μl/mouse), PM10 (30 ng/10 μl/mouse), administered daily and up to the time of mice sacrifice. To date, PM10, PM1, and Soot doses were chosen according to preliminary data during which mice were treated in a dose-dependent manner. The choice of the working dose was based on the effect on the lung in terms of inflammation, identified as positive to PAS staining (please refer below). In another group of mice, Ac-Y-VAD (Y-Vad, 10 μg/mouse), caspase-1 inhibitor, was intraperitoneally (i.p.) injected every 3 days, as already reported (8). Mice were divided into the following groups:

1. Vehicle, mice treated with DMSO 0.1%, which was used as Vehicle to dissolve Soot, PM1 and PM10, *n* = 8;
2. Soot, i.t. instilled with Soot, n = 8;
3. Soot+Y-Vad, i.t. instilled with Soot and Y-Vad, n = 8;
4. PM1, i.t. instilled with PM1, n = 8;
5. PM1+Y-Vad, i.t. instilled with PM1 and Y-Vad, n = 8;
6. PM10, i.t. instilled with PM10, n = 8;
7. PM10+Y-Vad, i.t. instilled with PM10 and Y-Vad, n = 8;
8. Sham-PBS treated mice, n = 5.

Mice were sacrificed at day 8, 14, and 28 following the first injection of Soot, PM1 or PM10. Left lung lobes were embedded into OCT medium to perform PAS staining to evaluate lung inflammation. Broncho-alveolar lavage fluid (BAL) was collected using 0.5 ml of PBS containing 0.5 mM EDTA to measure pro-inflammatory and anti-inflammatory cytokine levels.

### Preparation of Particulate Matter

Soot samples were collected from a laboratory flame, which was run in fuel-rich conditions feeding an ethylene/air mixture with an equivalence ratio Φ = 2.0 at atmospheric pressure. PM1 and PM10 samples were collected by means of an automatic outdoor station for continuous atmospheric particulate sampling (Tecora Skypost PM HV). The outdoor station was operated in a crowed area characterized by high automotive traffic during 2018 fall season and allowed to collect daily samples of particulate matter on filters. Particulate matter was later suspended in DMSO, following a sonication-assisted solvent extraction. Table 1 reports a summary of the elemental composition and size range of the investigated samples.

**Table 1:** Particle size and composition of PM_1_, PM_10_, and Soot samples.

	**PM_**1**_**	**PM_**10**_**	**Soot**
Particle size	<1 mm	<10 mm	<200 nm
C %	87.5	77	79.5
H %	n.d.	n.d.	19.5
O %	11.5	19.6	<1
S %	0.8	0.5	n.d.
K %	0.2	0.25	n.d.
Na %	n.d.	0.3	n.d.
Mg %	n.d.	0.3	n.d.
Al %	n.d.	0.3	n.d.
Ca %	n.d.	0.75	n.d.
Fe %	n.d.	0.25	n.d.
Si %	n.d.	0.75	n.d.

*n.d., not detected*.

Particle morphology and chemical composition were determined by Scanning Electron Microscopy analysis performed by a Hitachi TM3000 SEM, coupled with a built-in energy dispersive Xray detector SwiftED3000.

### PAS Staining

Left lung lobes were fixed in OCT medium (Pella Inc., Milan, and Italy) and 7 μm cryosections were cut. The degree of inflammation was scored by blinded observers by using Periodic acid/Alcian blue/Schiff staining (PAS). PAS Staining (Sigma Aldrich, Milan Italy) was performed according to the manufacturer's instructions to detect glycoprotein (9). PAS+ cryosections were graded with scores 0 to 4 to describe low to severe lung inflammation as follows: 0: <5%; 1: 5 to 25%; 2: 25–50%; 3: 50–75%; 4: <75% positive staining/total lung area.

### Airway Responsiveness Measurements

Bronchial rings of 1–2 mm length were cut and placed in organ baths mounted to isometric force transducers (Type 7006, Ugo Basile, Comerio, Italy) and connected to a Powerlab 800 (AD Instruments, Ugo Basile, Comerio, Italy). Rings were initially stretched until a resting tension of 0.5 g was reached and allowed to equilibrate for at least 30 min. In each experiment bronchial rings were challenged with carbacole in a concentration-dependent manner (1 pM−10 μM) in order to evaluate broncho-contraction. On the other hand, a cumulative concentration-response curve to salbutamol (10 pM−30 μM) on a stable tone of carbacole (1 μM) was performed in order to evaluate broncho-dilation.

### Flow Cytometry Analysis

Lungs were isolated and digested with 1 U/mL of collagenase (Sigma Aldrich, Rome, Italy). Cell suspensions were passed through 70 μm cell strainers, and red blood cells were lysed. To investigate the immune cells infiltrated into the lung of mice, lung cell suspensions were labeled with specific antibodies (CD11c, CD11b, Gr-1, F4/80, MHC II, CD80, Arginase I, CD3, CD4, CD25, and FoxP3).

### Cytokine Measurements

IL-1α, IL-1β, IL-33, IL-13, TNFα, IFN-⋎, and IL-10 were measured in BAL or lung homogenates as specified in the text, using commercially available ELISA kits (eBioscience, CA, USA). In the first case cytokine levels were expressed as pg/ml, whereas in lung homogenates as pg/mg protein. IL-1α, IL-1β, IL-33, IL-13, and TNFα cytokines were chosen to evaluate the pro-inflammatory milieu; whereas IFN-⋎ and IL-10 were chosen to define the adaptive immune environment.

### Statistical Analysis

Data are reported as median ± interquartile range and represented as box and whiskers. Statistical differences were assessed with TWO-WAY, ONE-WAY Analysis of variance (ANOVA) followed by multiple comparison post-tests as appropriate. Mann–Whitney *U*-test was performed where appropriate. *p* < 0.05 were considered significant.

## Results

### The Exposure of Mice to Particulate Matter (PM) Induces Lung Inflammation

**Figure 1:**
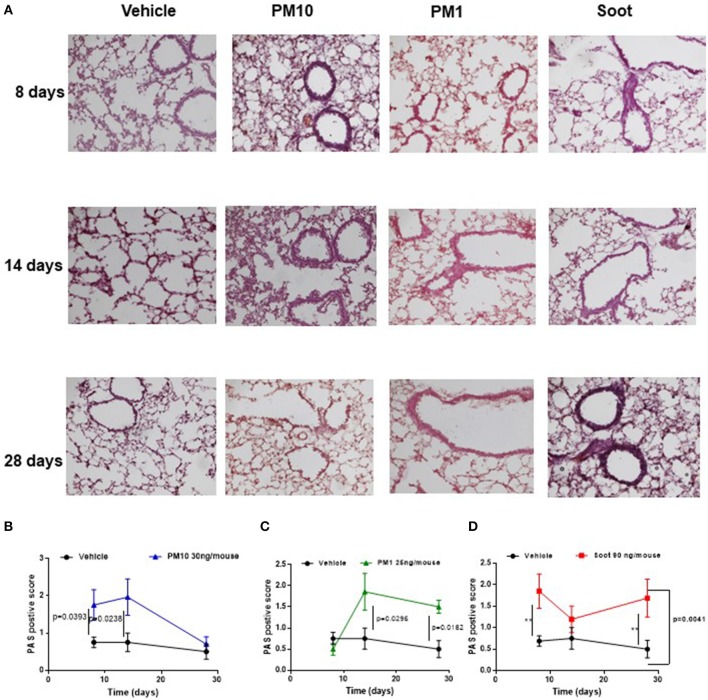
Exposure to PMx induced lung inflammation. Mice were intratracheally daily exposed to PM10 (30 ng/mouse, blue line), PM1 (25 ng/mouse, green line), and Soot (90 ng/mouse, red line). **(A)** PAS staining was performed on lung criosection derived from mice sacrificed at 8, 14, and 28 days after PM10, PM1, and soot exposure. **(B–D)** Quantitative analysis of PAS staining. Data are represented as mean ± SEM, *n* = 8. Two-Way ANOVA was performed and followed by Tukey's *post-hoc* test. *P* < 0.05 was considered significant.

It is well-known that PM can be differentiated according to the size (10). To this end, we collected different PMx, herein identified as PM10, particles with size smaller than 10 μm, as PM1 particles with size smaller than 1 μm and Soot, which represents the carbonaceous component of PM1 and PM10 (Table 1) (11). In order to evaluate the effect of these three fractions on the lung, mice were i.t. treated every day. The i.t. instillation of PM10 induced lung inflammation at 8 and 14 days post-treatment (Figures 1A,B, blue line) as determined by PAS staining (Figures 1A,B, blue line); whereas, at 28 days PAS staining did not highlight lung inflammation following PM10 treatment (Figures 1A,B, blue line) in that this group of mice had similar lung behavior as the Vehicle group (DMSO, 0.1%) (Figures 1A,B, black line). Similarly, the treatment with PM1 induced lung inflammation as higher PAS+ staining at 14 and 28 days (Figures 1A,C, green line), implying a delayed lung inflammation compared to PM10. Because both PM1 and PM10 comprise a carbonaceous component, herein defined as Soot, to evaluate whether anthropogenic fraction of PM, that related to automotive traffic, could be responsible for lung inflammation, mice were treated with Soot 90 ng/mouse. A marked hyperplasia around bronchi (Figure 1A) was associated with higher mucus production at 8 and 28 days, but not at 14 days, post-Soot treatment (Figure 1D, red line).

**Figure 2:**
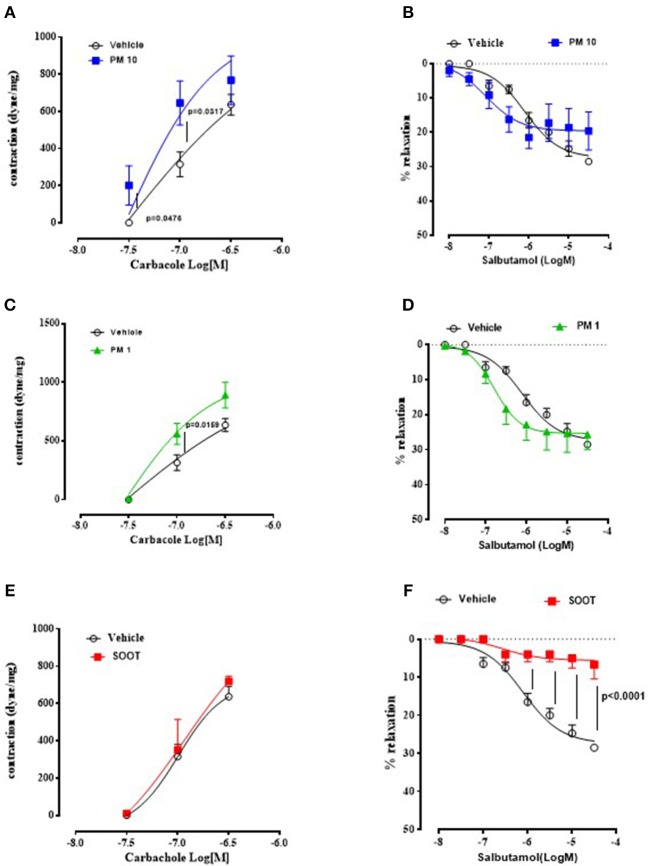
Exposure to PMx altered bronchial responsiveness. Bronchi from mice intratracheally exposed to PM10 (30 ng/mouse, blue line), PM1 (25 ng/mouse, green line), and Soot (90 ng/mouse, red line) were subjected to carbacol, bronchial contractant, and salbutamol, bronchial dilator. PM10 **(A)** and PM1 **(C)** increased carbacol responsiveness, compared to Soot-treated mice **(E)**. Instead, bronchial dilation was not altered in PM10 **(B)** and PM1 **(D)** treated mice, instead Soot-treated mice showed significantly reduced bronchial dilation to salbutamol **(F)**. Data are represented as mean ± SEM, *n* = 8. Two-way ANOVA was performed and followed by Tukey's *post-hoc* test. *P* < 0.05 was considered significant.

To prove bronchial dysfunction after PMx treatment, we measured airway responsiveness to carbacole and salbutamol. In the first case, we observed an alteration of the bronchial tone following a cumulative administration of carbacole in PM10 (Figure 2A) and PM1 (Figure 2C) group of mice compared to the Vehicle group (Figures 2A,C, black line). In particular, PM10- (Figure 2A, blue line) and PM1-treated (Figure 2C, green line) mice showed bronchial responsiveness starting from 0.1 μM of carbacole compared to the Vehicle group (Figures 2A,C, black line). In contrast, mice treated with Soot did not show any alteration of the bronchial tone compared to the Vehicle (Figure 2E). Interestingly, bronchi from PM10- (Figure 2B) and PM1-treated (Figure 2D) mice did not have an altered broncodilation under a concentration-dependent challenge of salbutamol; instead, bronchi from Soot-treated mice showed a robust reduction of dilation under salbutamol challenge compared to the Vehicle group (Figure 2F).

Taken together, these data suggest that PMx induce lung inflammation in mice, although in a different mode in that they all induce mucus hypersecretion (PAS + staining) but the smallest component, Soot (20–40 nm) can damage the epithelium altering the bronchial physiological dilation.

### The Exposure of Mice to Particulate Matter (PM) Induces Lung Immunosuppression

**Figure 3:**
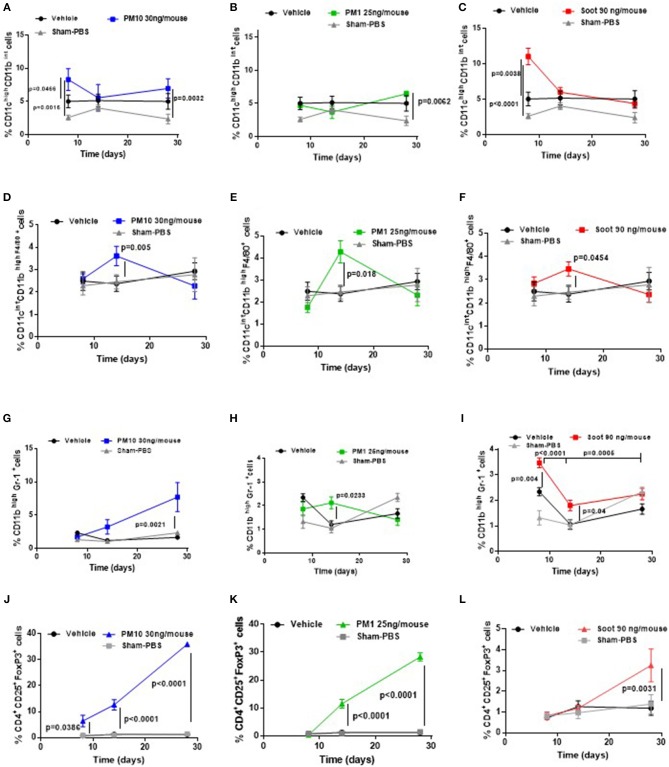
Exposure to PMx altered lung immune microenvironment in a time-dependent manner. Lungs from mice intratracheally exposed to PM10 (30 ng/mouse, blue line), PM1 (25 ng/mouse, green line), and Soot (90 ng/mouse, red line) were digested with Collagenase and flow cytometry analysis was performed. PM10 **(A)** and Soot **(C)**, but not PM1 **(B)** induced higher recruitment of DCs at early time point. Similarly, PM10 **(D)**, PM1 **(E)**, and Soot **(F)** induced higher recruitment of macrophages at 14 days. Higher recruitment of MSDCs was observed after PM10 **(G)**, PM1 **(H)**, and Soot **(I)** treatment. This effect was associated to higher presence of Treg after PM10 **(J)**, PM1 **(K)**, and Soot **(L)** exposure. Data are represented as mean ± SEM, *n* = 8. Two-Way ANOVA was performed and followed by Tukey's *post-hoc* test. *P* < 0.05 was considered significant.

Based on our previous published data, the administration of PMx can alter immune cell phenotype (6, 7). Therefore, we evaluated immune cell infiltration in the lung of the five groups of mice. The administration of PM10 significantly increased the recruitment of dendritic cells (DCs, identified as CD11c^high^ CD11b^int^ F4/80^neg^) at 8 days (Figure 3A, blue line), of macrophages (identified as CD11c^int^ CD11b^high^ F4/80^pos^) at 14 days (Figure 3D, blue line), of myeloid-derived suppressor cells (MDSCs, identified as CD11b^high^ Gr-1^pos^) at 14 and 28 days (Figure 3G, blue line) and of T regulatory cells (Treg, identified as CD3^+^CD25^+^CD4^+^FoxP3^+^) at 14 and 28 days (Figure 3J, blue line) compared to the Vehicle and Sham group (Figure 3, black and gray lines). Similarly, the lungs of PM1-treated mice had significantly higher percentage of recruited macrophages at 14 days (Figure 3E, green line), MDSCs at 14 days (Figure 3H, green line) and Treg at 14 and 28 days post-treatment (Figure 3K, green line) compared to the Vehicle group (Figure 3, black line). No differences were observed with the recruitment of DCs (Figure 3B, green line) compared to the Vehicle and Sham group (Figure 3B, black and gray line).

Interestingly, Soot particles had similar behavior to PM10 and PM1 in that they significantly increased the percentage of DCs (Figure 3C, red line) at 8 days, of macrophages at 14 days (Figure 3F, red line), of MDSCs at 14 days (Figure 3I, red line) and of Treg at 28 days (Figure 3L, red line) compared to the Vehicle and Sham group.

**Figure 4:**
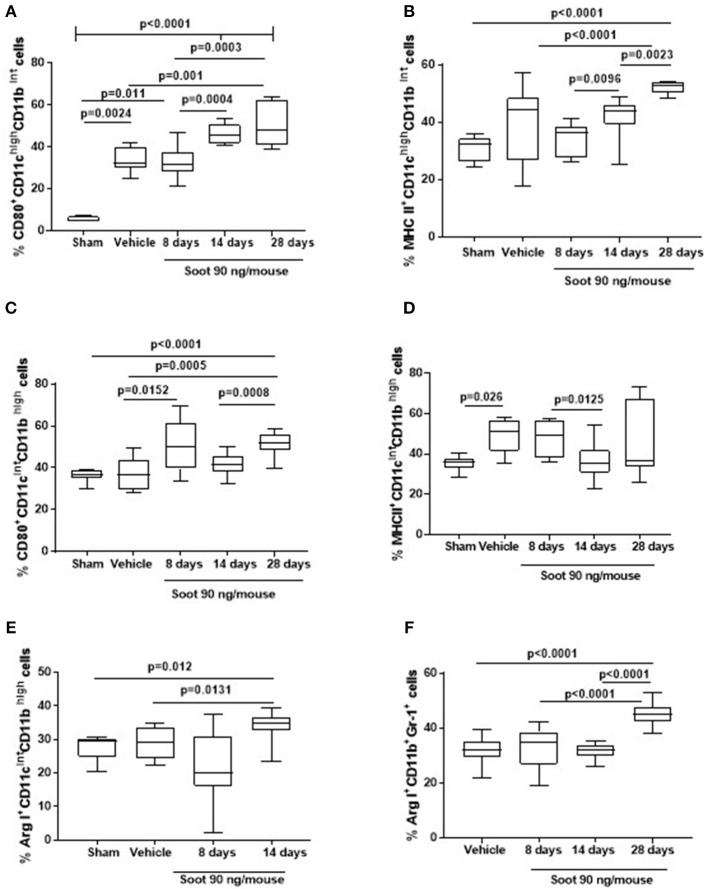
Exposure to Soot altered innate immune cell phenotype recruited to the lung in mice. Soot-treated mice showed higher levels of CD80 **(A)**, but not MHC II **(B)** on DCs. Similar effect was observed for macrophages regarding CD80 **(C)** but not MHC II **(D)** expression. Macrophages **(E)** and MSDCs **(F)** recruited to the lung of Soot-treated mice showed significantly higher levels of Arginase I. Data are represented as mean ± SEM, *n* = 8. One-Way ANOVA was performed and followed by Dunn's *post-hoc* test. *P* < 0.05 was considered significant.

To understand the phenotype of the innate immune cells, we analyzed the expression of CD80 and MHC II on both DCs and macrophages. The administration of Soot significantly increased the levels of CD80 (Figure 4A), but not of MHC II (Figure 4B) on DCs. To note, Sham mice had very low levels of CD-80 than the Vehicle group (Figure 4A). Similarly, macrophages presented higher levels of CD80 (Figure 4C) but lower levels of MHC II (Figure 4D) compared to the Vehicle and Sham group. Moreover, macrophages had higher levels of the immunosuppressive Arginase I in Soot-treated mice compared to the Vehicle group (Figure 4E). In the same manner, Arginase I was significantly higher in lung MDSCs from Soot-treated mice than Vehicle and Sham group (Figure 4F).

Similar data was observed for PM10- and PM1-treated mice in that both macrophages (Vehicle: 27.1 ± 2.6 vs. PM10: 48.5 ± 3.6 or PM1: 42 ± 3.04) and MDSCs (Vehicle: 32.1 ± 1.16 vs. PM10: 59.3 ± 1.43 or PM1: 55.17 ± 1.06) presented higher levels of the immunosuppressive Arginase I.

**Figure 5:**
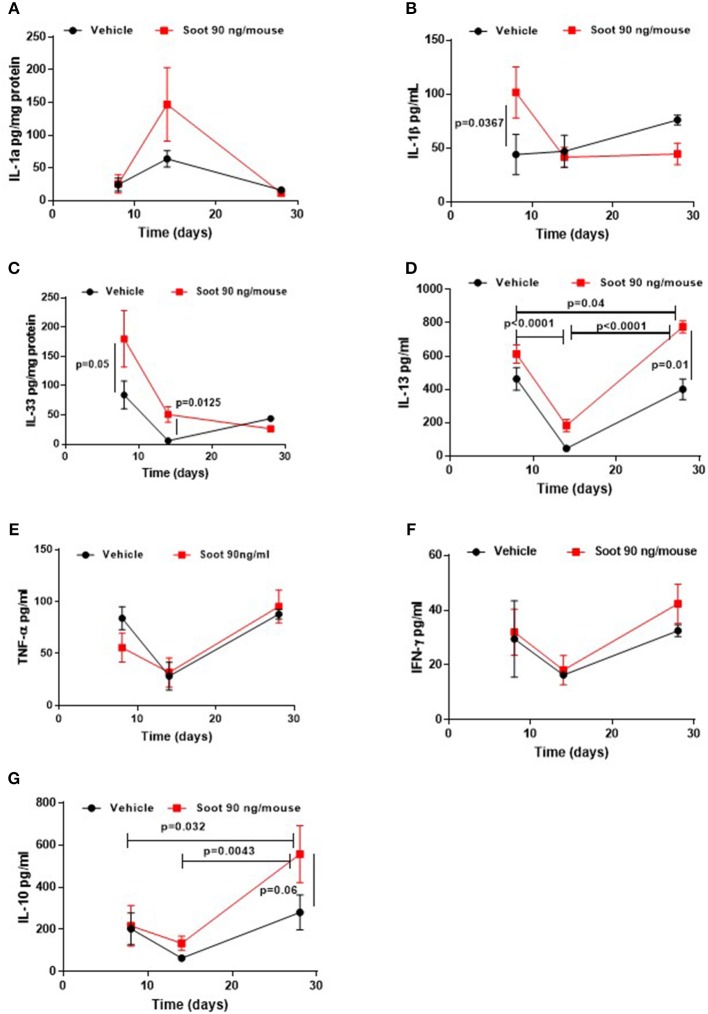
Exposure to Soot altered cytokine signature into the lung of mice. IL-1a **(A)** and IL-33 **(C)** were analyzed in a time-dependent manner in lung homogenates obtained from Soot-treated mice. IL-1b **(B)**, IL-13 **(D)**, TNFa **(E)**, IFN⋎ **(F)**, and IL-10 **(G)** were tested in the BAL of Soot-treated mice. Data are represented as mean ± SEM, *n* = 8. Two-Way ANOVA was performed and followed by Tukey's *post-hoc* test. *P* < 0.05 was considered significant.

To further evaluate the immune microenvironment, pro- and anti-inflammatory cytokine levels were analyzed. Soot-treated mice showed a slight increase of IL-1α levels at 14 days (Figure 5A), whereas PM10- (Supplementary Figure 1A) and PM1- (Supplementary Figure 2A) treated mice showed a significant increase in this cytokine at later time points, probably due to the presence of other than carbonaceous components such as metals and oxides. Soot-, similarly to PM10- (Supplementary Figure 1B) and PM1- (Supplementary Figure 2B), treated mice had higher levels of IL-1β levels at earlier time points (Figure 5B). Similarly, IL-33 levels were higher at earlier time points in Soot-treated mice (Figure 5C) compared to the Vehicle group. PM10- (Supplementary Figure 1C) and PM1-treated (Supplementary Figure 2C) mice had higher levels of IL-33 starting from 8 days up to 28 days of treatment, probably due to the presence of non-carbonaceous components. Soot-treated (Figure 5D), differently from PM10- (Supplementary Figure 1D) and PM1-treated (Supplementary Figure 2D), mice had higher levels of IL-13, explaining the reduced bronchial dilation observed in Figure 2F. Instead, no differences were noted for TNFα and IFN⋎ in the lung of Soot-treated (Figures 5E,F, respectively) and of PM10- (Supplementary Figures 1E,F) and PM1-treated (Supplementary Figures 2E,F) mice.

Interestingly, IL-10 levels were significantly higher in Soot- (Figure 5G), PM10- (Supplementary Figure 1G) and PM1-treated mice (Supplementary Figure 2G), further confirming the immunosuppressive lung microenvironment.

### Soot-Induced Immunosuppressive Lung Microenvironment Was Not Dependent on Caspase-1

**Figure 6:**
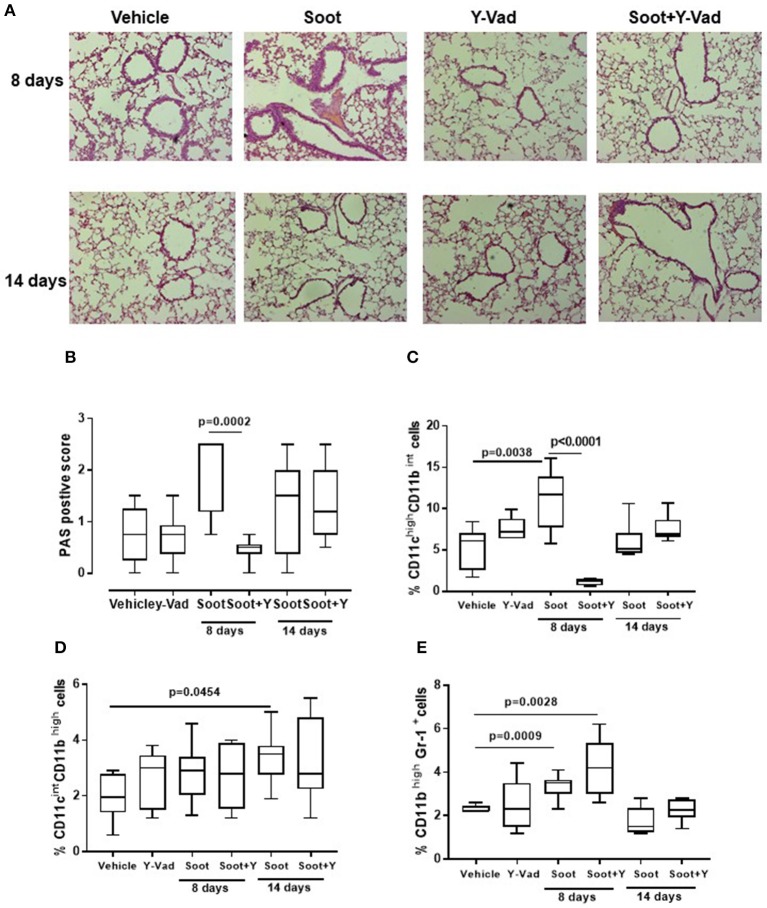
Exposure to Soot induced lung inflammation in a caspase-1-indepdent manner. Mice exposed to Soot showed that the inhibition of caspase-1 was able to reduce PAS staining at the sole early time point (8 days) **(A,B)**. In support, DCs **(C)**, macrophages **(D)**, and MDSC **(E)** were still recruited to the lung in Soot+Y-Vad-treated mice. Data are represented as mean ± SEM, *n* = 8. Two-Way ANOVA was performed and followed by Tukey's *post-hoc* test. *P* < 0.05 was considered significant.

In our previous data (6, 7), we found that the release of IL-1-like cytokines from Soot-treated human cells was correlated to the inflammasome complex. Therefore, because caspase-1 is involved in the inflammasome activation and in IL-1-like cytokine release (12, 13), we treated mice with a well-known caspase-1 inhibitor, Ac-Y-Vad, Y-Vad. The inhibition of caspase-1 significantly reduced PAS positive staining at 8, but not at 14 days post-Soot treatment (Figures 6A,B). Bronchial hyperplasia was not evident in Soot+Y-Vad-treated mice compared to Soot-treated mice at 8 days (Figure 6A). Nevertheless, this effect was not time-dependent (Figures 6A,B). Similar data was observed for PM10- and PM1-treated mice (Supplementary Figures 3A,B).

In support, the recruitment of DCs was decreased at the sole 8 days but not at later time points (Figure 6C) after Soot treatment. To date, the inhibition of caspase-1 did not alter the recruitment of macrophages (Figure 6D) and MDSCs (Figure 6E) after Soot treatment. In the same manner, the levels of IL-1α (Supplementary Figure 4A), IL-33 (Supplementary Figure 4C) and IFN⋎ (Supplementary Figure 4D) were not altered in Soot+Y-Vad- vs. Soot-treated mice. Instead, the levels of IL-1β (Supplementary Figure 4B) were decreased at the sole 8 days in Soot+Y-Vad group of mice, implying that the involvement of caspase-1 in lung inflammation is at early time points and that it does not play a pivotal role for the induction of late lung inflammation after PM exposure.

## Discussion

In the present study, we found that PM-exposed mice were susceptible to lung inflammation which was not characterized by an acute pro-inflammatory, but rather by an immunosuppressive environment.

PM10, PM1, and Soot were used as examples of particles mimicking air pollutants in that PM10 represents the entire class of inhalable atmospheric particles; PM1 represents the inhalable particulate matter whereas Soot is representative of the carbonaceous material of anthropogenic origin emitted from engines. The main difference between Soot and PMx (PM10 or PM1) was the presence of inorganic components such as metals, sulfur and nitric oxide (Table 1), factors comprising the ozone-induced secondary aerosol. Besides metals, our data suggest that the carbonaceous core of PM, Soot, is pivotal for the induction of lung inflammation. Regarding the term “lung inflammation” we have to point at the fact that we did not observe a pro-inflammatory environment, rather, we observed an immunosuppressive environment. Indeed, the exposure of mice to PM10, PM1, and Soot increased the recruitment of innate immune cells such as DCs and macrophages, but these latter were in their immunosuppressive phenotype. DCs as well as macrophages presented higher levels of CD80 but lower levels of MHC II, which translated in a tolerogenic nature of these cells in that they were not able to induce a cytotoxic and effective adaptive immunity (14, 15). Indeed, both IFN⋎ (Supplementary Figure 1) and CD4^+^ (Vehicle: 21.33 ± 2.4 vs. PM10: 29.9 ± 0.6 or PM1: 25.6 ± 1.6) and CD8^+^ (Vehicle: 11.33 ± 0.8 vs. PM10: 9.8 ± 1.06 or PM1: 15.3 ± 0.96) T cells were not increased in PM-exposed mice compared to the Vehicle group. Instead, we observed that macrophages and MDSCs, which were significantly increased in the lung of PM-exposed mice, were positive to Arginase I, which is an enzyme involved in the metabolism of L-Arginine, which is converted into urea and L-ornitine (16). This metabolic reaction in innate immune cells is expression of a Th2- or Treg-preferred immunosuppressive environment which translates in an anti-inflammatory, tolerogenic activity (16, 17). In this scenario, PMx and Soot induced recruitment of immunosuppressive DCs, macrophages and MDSCs was associated to the higher presence of Treg and higher levels of IL-10 (18), which render the lung less susceptible to acute inflammatory-driven damages, but pave the way for higher susceptibility to other respiratory disorders, such as fibrosis and, even worse, cancer (19). In support, it is well-known that the immune system plays a pivotal role in tumor immune escape in that the immune cells are tolerogenic and therefore incapable of recognizing transformed cells as non-self (20). In this context, immunosuppression overtakes the immunostimulatory activity of innate immune cells such as DCs and macrophages, which in their tolerant phenotype can facilitate the immunosuppressive arm of the adaptive immune system (21). Several epidemiological studies have demonstrated that there is a direct link between lung cancer and air pollution, although little is known about the cellular and molecular underlying mechanism (22). In this study, we found for the first time, to our knowledge, that the exposure of mice to air pollutants can create an immunosuppressive environment that could favor several respiratory disorders. However, we have to point at a limitation to our study which lacks of the identification of the molecular sensor for PMx, which still needs further studies.

Another issue that needs to be pointed at is the airway responsiveness of mice exposed to the three types of air pollutants we used. We found that the sole Soot significantly reduced bronchodilation following salbutamol, a β2 adrenergic agonist, which is clinically used as an anti-asthmatic as well as in long-acting β2 adrenergic treatment of COPD patients (23). It has to be noted that a limitation to our experimental conditions was the intratracheal exposure that does not entirely mimic the physiological exposure to air pollution which is usually suspended in the air as aerosol. However, despite the limitation about the intratracheal instillation, the reduction of bronchodilation was observed for the sole Soot most likely due to the particle size that were instilled compared to PM10 and PM1, which were of bigger size. In support, in our previous study we found that peripheral blood cells of COPD patients, as well as smokers, were more susceptible to Soot exposure in that they released higher levels of IL-1-like cytokines, which can participate in an inflammatory context to lung damage (6). It still remains, though, to elucidate whether Soot-induced epithelial damage was associated to IL-1-like cytokines, implying lower dilation of bronchi, typical of asthma, COPD and other respiratory disorders. Nevertheless, the cross-talk between our previous data on human primary cells and the actual data on an animal model is the level of IL-1-like cytokines (i.e., IL-1α, IL-1β, and IL-33), which are related to the inflammasome complex activation, most likely associating lung immunosuppression to pro-fibrotic processes, as observed in other respiratory diseases (24, 25).

Therefore, taking advantage of the pre-clinical model, we used a pharmacological inhibitor of caspase-1, the enzyme which conveys into the inflammasome complex and that is able to auto-cleave, promoting IL-1-like cytokine release (12, 13). Interestingly, we found that the inhibition of caspase-1 did not alter the recruitment of DCs, macrophages and MSDCs to the lung of PM-exposed mice. Moreover, PM-induced immunosuppression was not modified by the pharmacological inhibition of caspase-1, implying that the canonical inflammasome complex is not involved. Similarly, in our previous data, we found that caspase-1 was not responsible for Soot-induced IL-1 release in COPD-derived PBMCs (6), confirming that either the non-canonical inflammasome is involved (26, 27) or that differential molecular mechanism/s can be induced after PM exposure.

## Conclusion

This study proves that PM exposure leads to an immunosuppressive environment in the lung and that this effect is not associated to the canonical inflammasome, caspase-1-dependent pathway. These studies can pave the way to understand the molecular and cellular mechanisms underlying the establishment of respiratory disorders according to air pollution exposure.

## Data Availability

All datasets generated for this study are included in the manuscript and/or the Supplementary Files.

## Ethics Statement

All animal experiments were performed under protocols that followed the Italian and European Community Council for Animal Care (2010/63/EU). This study was carried out in strict accordance with the recommendations in the Guide for the Care and Use of Laboratory Animals of the National Institutes of Health. The protocol was approved by the Committee on the Ethics of Animal Experiments of the University of Salerno and Health Ministry with the approval number 985/2017.

## Author Contributions

CC, GD, MT, FR, IC, and MS performed experiments. GC, RA, AD, and AP read and edited the manuscript. RS designed the experiments, analyzed and interpreted the data.

### Conflict of Interest Statement

The authors declare that the research was conducted in the absence of any commercial or financial relationships that could be construed as a potential conflict of interest.

## Supplementary Material

Supplementary Figure 1 Cytokine levels after PM10 exposure. IL-1a **(A)** and IL-33 **(C)** were analyzed in a time-dependent manner in lung homogenates obtained from PM10-treated mice. IL-1b **(B)**, IL-13 **(D)**, TNFa **(E)**, IFN⋎ **(F)**, and IL-10 **(G)** were tested in the BAL of PM10-treated mice. Data are represented as mean±SEM, *n* = 8. Two-Way ANOVA was performed and followed by *post-hoc* test. *P* < 0.05 was considered significant.

Supplementary Figure 2 Cytokine levels after PM1 exposure. IL-1a **(A)** and IL-33 **(C)** were analyzed in a time-dependent manner in lung homogenates obtained from PM1-treated mice. IL-1b **(B)**, IL-13 **(D)**, TNFa **(E)**, IFN⋎ **(F)**, and IL-10 **(G)** were tested in the BAL of PM1-treated mice. Data are represented as mean ± SEM, *n* = 8. Two-Way ANOVA was performed and followed by *post-hoc* test. *P* < 0.05 was considered significant.

Supplementary Figure 3 PAS positive staining analyzed in a time dependent manner in PM1 **(A)** and PM 10 **(B)** treated mice in the presence or not of y-VAD. Two-Way ANOVA was performed and followed by *post-hoc* test. *P* < 0.05 was considered significant.

Supplementary Figure 4 Cytokine levels after Soot exposure. IL-1a **(A)** and IL-33 **(C)** were analyzed in a time-dependent manner in lung homogenates obtained from Soot-treated mice. IL-1b **(B)** and IFN⋎ **(D)** were tested in the BAL of Soot-treated mice in the presence of y-VAD. Data are represented as mean ± SEM, *n* = 8. Mann Whitney *U*-test was performed.

## Abbreviations

WHO: World Health Organization
PM: particulate matter
UFPs: ultrafine particles
MDSCs: myeloid-derived suppressor cells
DCs: dendritic cells
Treg: T regulatory cells.
