# Supplementary figures and images for: The Inhibition of Caspase-1- Does Not Revert Particulate Matter (PM)-Induced Lung Immunesuppression in Mice
Source: Front Immunol. 2019 Jun 21;10:1329. doi: 10.3389/fimmu.2019.01329 (PMC6598547; doi:10.3389/fimmu.2019.01329)

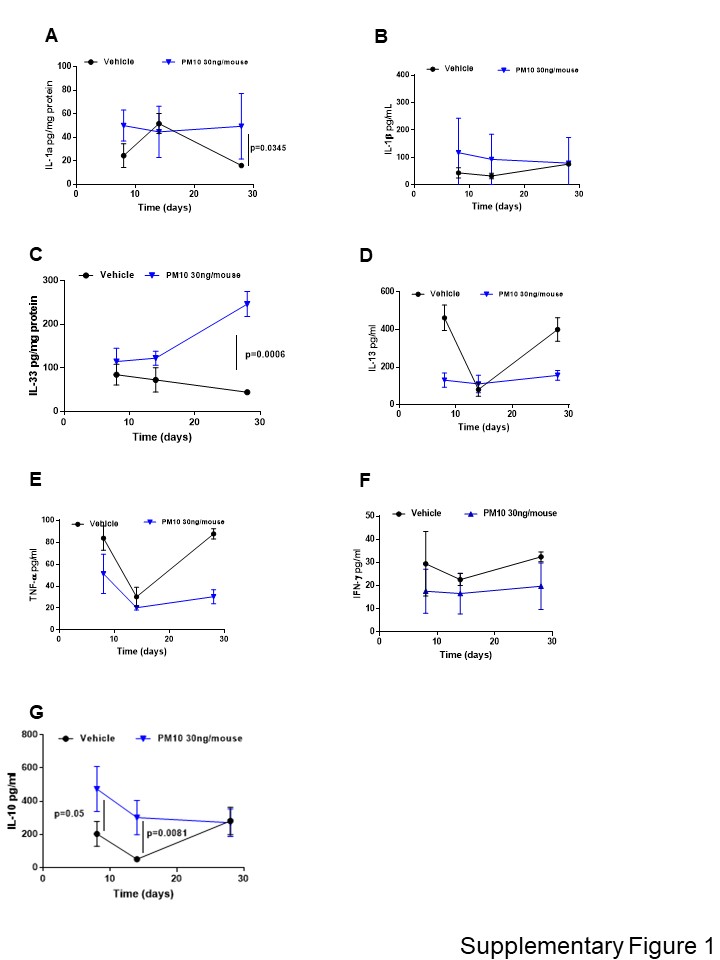

Supplement: Supplementary Figure 1 — Cytokine levels after PM10 exposure. IL-1a (A) and IL-33 (C) were analyzed in a time-dependent manner in lung homogenates obtained from PM10-treated mice. IL-1b (B), IL-13 (D), TNFa (E), IFN⋎ (F), and IL-10 (G) were tested in the BAL of PM10-treated mice. Data are represented as mean±SEM, n = 8. Two-Way ANOVA was performed and followed by post-hoc test. P < 0.05 was considered significant. [file Image_1.JPEG]

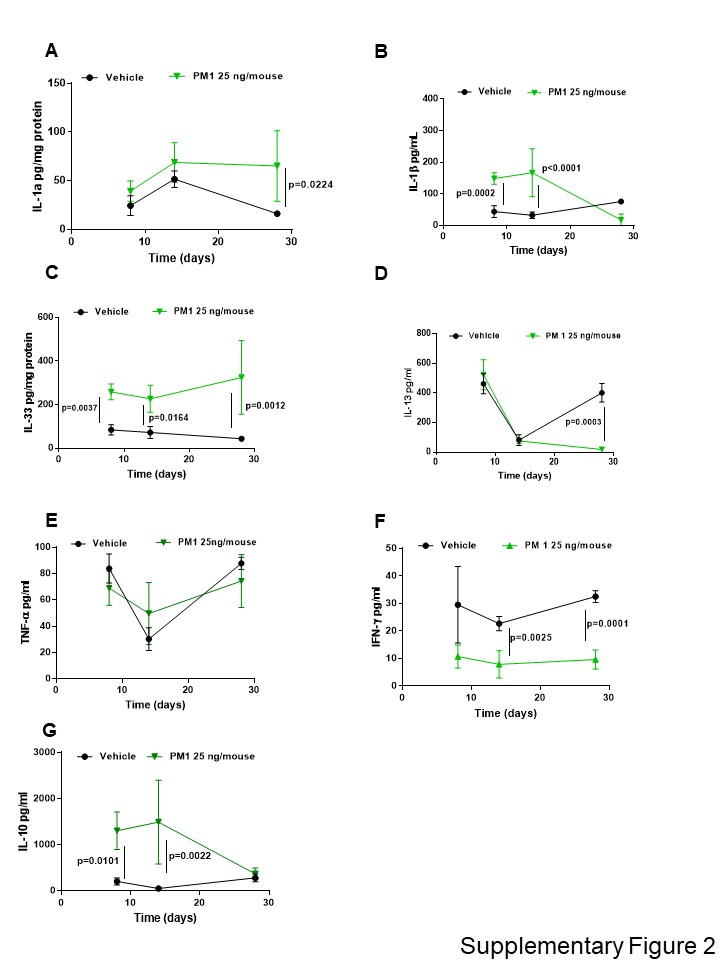

Supplement: Supplementary Figure 2 — Cytokine levels after PM1 exposure. IL-1a (A) and IL-33 (C) were analyzed in a time-dependent manner in lung homogenates obtained from PM1-treated mice. IL-1b (B), IL-13 (D), TNFa (E), IFN⋎ (F), and IL-10 (G) were tested in the BAL of PM1-treated mice. Data are represented as mean ± SEM, n = 8. Two-Way ANOVA was performed and followed by post-hoc test. P < 0.05 was considered significant. [file Image_2.JPEG]

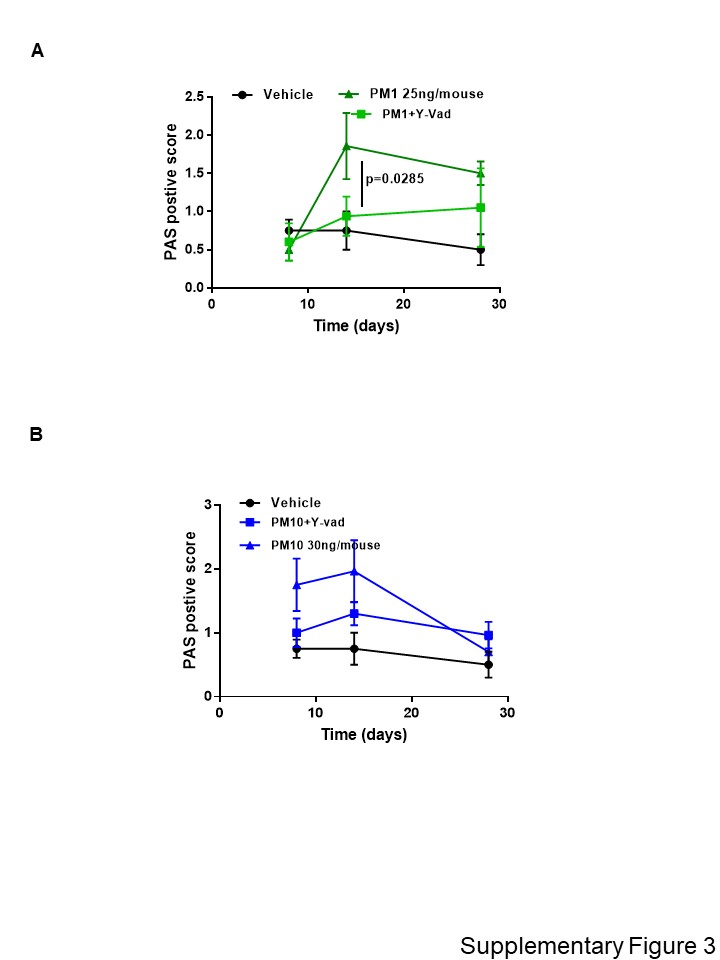

Supplement: Supplementary Figure 3 — PAS positive staining analyzed in a time dependent manner in PM1 (A) and PM 10 (B) treated mice in the presence or not of y-VAD. Two-Way ANOVA was performed and followed by post-hoc test. P < 0.05 was considered significant. [file Image_3.JPEG]

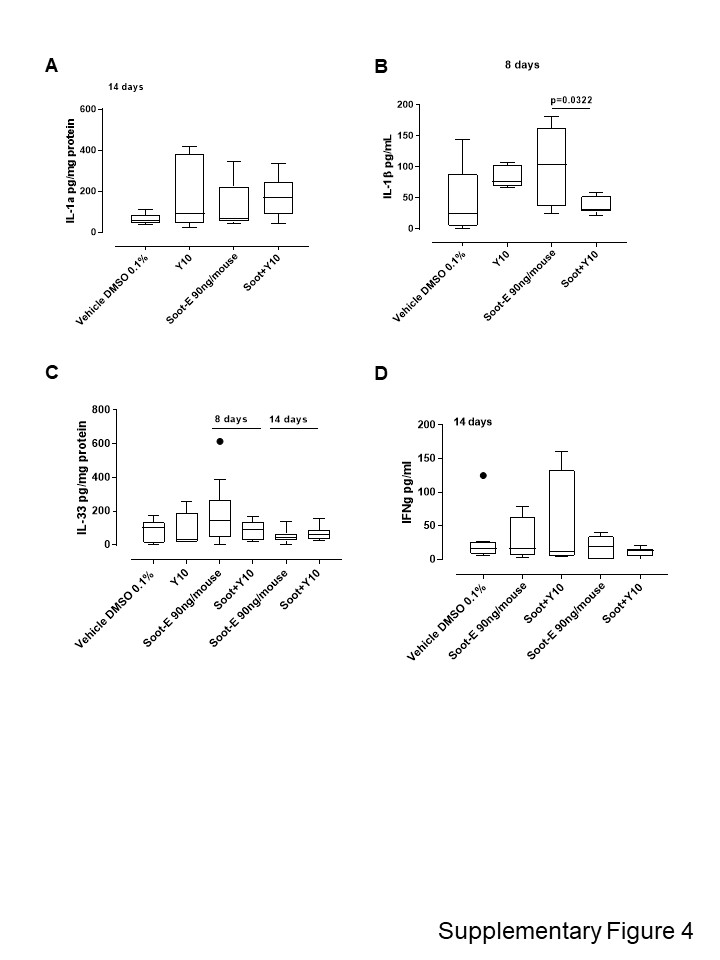

Supplement: Supplementary Figure 4 — Cytokine levels after Soot exposure. IL-1a (A) and IL-33 (C) were analyzed in a time-dependent manner in lung homogenates obtained from Soot-treated mice. IL-1b (B) and IFN⋎ (D) were tested in the BAL of Soot-treated mice in the presence of y-VAD. Data are represented as mean ± SEM, n = 8. Mann Whitney U-test was performed. [file Image_4.JPEG]
